# The Virginia Medical Monthly

**Published:** 1874-04

**Authors:** 


*The Virginia Medical Monthly.*

This is the title of a new journal devoted to medical science and its literature, and published by Dr. Landon B. Edwards, editor and proprietor. We are in receipt of the first number. It gives excellent evidences of permanent success. From our personal acquaintance and knowledge of the editor, we can safely affirm that the *Virginia Medical Monthly* will be conducted in a manner alike creditable to the fine medical and literary attainments of its founder, and the State whose honored title the journal bears.

Virginia, in its medical profession, as in all the branches of science, art, literature and statesmanship that add lustre to civilization, stands in the foremost rank, and we hope that our medical brethren of the Old Commonwealth will come forth to the support of this new candidate for public favor, and from their abundance of medical lore and fund of scholastic research in the vast field of physical investigation, contribute their quota to its columns. A model medical journal should be the pride of the profession, and invigorated by the vital spirit of progress and moved by the genial rays of intellectuality. With these views, we welcome the *Virginia Medical Monthly*, the newly born but promising relative ot the SOUTHERN MEDICAL RECORD, and bid it godspeed every where in its labor of love and career of usefulness with their mutual personal friends and true lovers of truth and science. T. S. P.

